# The role of the season at admission in neonatal sepsis: a retrospective chart review of a 1-year data at University of Gondar comprehensive specialized hospital

**DOI:** 10.1186/s13104-019-4685-2

**Published:** 2019-10-04

**Authors:** Temesgen Worku Gudayu, Ejigu Gebeye Zeleke, Ayenew Molla Lakew

**Affiliations:** 1 Department of Clinical Midwifery, School of Midwifery, College of Medicine and Health Sciences, University of Gondar, Gondar, Ethiopia; 2 Department of Epidemiology and Biostatistics, Institute of Public Health, College of Medicine and Health Sciences, University of Gondar, Gondar, Ethiopia

**Keywords:** Neonatal sepsis, University of Gondar, Early-onset neonatal sepsis

## Abstract

**Objective:**

Neonatal sepsis is a global public health concern in general and causes a massive burden in developing countries particularly in sub-Saharan Africa. Though it is mostly preventable, neonatal sepsis remained the leading cause of mortality in developing countries. This study was conducted to determine the current proportion and identify factors associated with neonatal sepsis to suggest directions.

**Results:**

In this study 504 randomly selected neonatal charts were reviewed. The proportion of overall neonatal sepsis was 63.69% (95% CI 59.38, 67.79), where early-onset sepsis was 59.33% (95% CI 54.96, 63.55) and late-onset sepsis was 4.17% (95% CI 2.73, 6.31). Maternal intra-partum fever, season of birth and admission, vaginal mode of delivery and preterm gestational age at birth increased the likelihood of overall and early-onset neonatal sepsis. In conclusion of this study, neonatal sepsis remaining the leading cause of morbidity among younger infants. Intra-partum conditions were major contributors to neonatal sepsis. Thus, providing emphasis on associated factors in particular and universal safe obstetric care in general is recommended.

## Introduction

Globally, an estimated 2.8 million neonatal deaths occurred in 2013 and the mortality showed a rising trend from 2000 to 2013 [1]. Among the death of under-5 years old children, neonatal death increased from about 37% in 2000 to nearly 42% in 2013 [1, 2].

Neonatal mortality in sub-Saharan Africa is estimated to be 29 per 1000 live births [3]. Similarly, Ethiopia is struggling with 29 per 1000 live births. The distribution of neonatal mortality throughout the country varies from region to region; 18 in Addis Ababa, 34–38 in other regions, and 47 in Amhara region that put Amhara region the highest neonatal death section in the country [4].

According to the 2016 report, 2.6 million children death occurred in the first month of life and the first week of life holds the highest proportion [5]. Prematurity, intra-partum related events such as birth asphyxia and birth trauma, and neonatal sepsis together contributed to almost three-quarters of all neonatal deaths [6].

A systematic infection, neonatal sepsis, is an important cause of morbidity and mortality of neonates. It resulted in 7% of loss and is the third leading cause of death in the globe [1, 7]. Previous reports indicated that yearly cases of neonatal sepsis in sub-Saharan Africa ranged from 380,000 to 2,000,000 with annual related neonatal deaths of 270,000 [8, 9].

In developing countries due to the scarcity of microbiological investigations; a clinical criteria in identifying neonates with possible sepsis are commonly used approach. Sepsis in neonates is commonly described as a clinical syndrome and distinguished by signs and symptoms. Fever, temperature instability, vomiting, diarrhea, irritability, lethargy, breathing problem, low blood sugar, jaundice, reduced sucking, and seizures are the most common symptoms of neonatal sepsis [10].

In Ethiopia, some studies showed that clinically diagnosed neonatal sepsis was the common neonatal morbidity with a proportion in excess of 75% [11, 12]. Also the bacterial pathogen isolation rate in neonatal sepsis was high and almost 70% of isolates were multiple drug-resistant strains [13].

It is reported that neonatal, infant and under-five mortality in general is inversely related to the gross domestic product per capita purchasing power, particularly of developing countries [14]. Specifically, neonatal sepsis poses an enormous public health burden for the sub-Saharan Africa with a significant associated economic cost. So that strategic investment in illustration of the disease etiology, diagnosis, treatment, and prevention is recommended [15].

This study, therefore, intended to determine the current proportion of clinically diagnosed neonatal sepsis at the University of Gondar Comprehensive Specialized Hospital. The study also aimed to identify factors associated with neonatal sepsis so that practitioners and policymakers working at the neonatal health settings will consume the findings.

## Main text

### Methods

A retrospective study was conducted among neonates admitted in an intensive care unit (NICU) from January 1st to December 31st, 2017 at the University of Gondar Comprehensive Specialized Hospital. The Hospital is located at about 740 km northwest of Addis Ababa and as part of the country the area has four seasons. Summer is a rainy season extends from June to August characterized by heavy rain falls. September to November is a spring season which is characterized by a dry and moist weather. Winter, a dry and sunny season runs from December to February and autumn runs from March to May [16].

The data were extracted from April 16 to May 15, 2018, at the University of Gondar Specialized Hospital record room. The Hospital is found in Gondar town and serves for more than seven million people residing in the northwest part of the country. The neonatal intensive care unit (NICU) is a unit under the pediatrics and child health department and the unit provides an inpatient medical service for neonates. It has a caring capacity of about 30 beds at a time. Averages of nearly 2500 neonates were admitted in the unit between January 1st and December 31st, 2017.

Neonates admitted in the intensive care unit of the University of Gondar Comprehensive Specialized Hospital from January 1st to December 31st, 2017 were included in this study. Whereas, those neonatal charts which had an incomplete observation of major variables such as admission date, diagnosis and outcome were excluded.

The sample size was determined using Epi-info version 7.2 statistical software; Statcalc for cohort or cross-sectional study sample size calculator. We considered the assumptions: 95% confidence level, 80% power, unexposed to the exposed ratio of 1, 86.77% outcome in the unexposed groups, the odds ratio of 2.859 [12] and 15% contingency. Then, a sample size of 504 was calculated. Finally, the sample size was distributed proportional to each month based on an estimated caseload and then a simple random sampling technique was used to select neonatal charts.

A clinically diagnosed neonatal sepsis was used as an outcome variable in this study. Explanatory variables such as maternal socio-demographic, obstetric and neonatal variables were also extracted from the chart.

A semi-structured data extraction tool was used for data extraction. It was prepared in English and data extraction was done by trained data extractors. The data extraction process was evaluated daily and necessary adjustment was made. During the data extraction period, about 120 randomly selected charts were incomplete, missed major variables and replaced by new random samples.

The collected data were checked manually for completeness and entered into Epi-info software version 7.2. The data were transported to STATA version 14 for analysis. The descriptive analysis was done and results were presented in tables and text form.

Bivariate logistic regression analysis was done for each explanatory variable with the outcome variable, clinically diagnosed neonatal sepsis. Independent variables that were statistically associated with the outcome variable at a *P* value of 0.2 in the bivariate analysis were further run in the multivariable logistic regression model. The model fitness was assessed by post estimation Pearson or Hosmer–Lemeshow goodness-of-fit test at a P-value of > 0.05. Then the final associated factors were determined using the adjusted odds ratio with its 95% confidence level and statistical significance was declared at P-value of ≤ 0.05.

### Results

#### Maternal characteristics of neonates admitted in the NICU

A total of 504 charts of neonates admitted in the NICU were reviewed. The median age of the mothers was 26 (IQR: 9) years and more than three quarters (78.17%) of them were between the age of 20 and 35 years. More than half, (56.55%), of the mothers resided out of Gondar town. The majority, (96.22%), of mothers attended ANC and (88.57%) of them were received TT^2+^ during their index pregnancy. About 157 (31.16%) of the mothers had at least one complication during index pregnancy and premature rupture of membrane (PROM) occurred in 13% of cases.

Approximately, about 80% of the mothers had a singleton pregnancy and in about 82% of the mothers the labor was of spontaneous onset. More than two-third (65.08%), of mothers gave spontaneous vaginal delivery and about 73% births occurred at Hospitals (Table 1).

**Table 1:** Socio-demographic and obstetric characteristics of mothers of neonates admitted in NICU of the UoGCSH from January to December 2017, northwest Ethiopia (n = 504)

Characteristics	Frequency	Percent
Current maternal age in years		
< 20	51	10.12
20–34	394	78.17
≥ 35	59	11.71
Residence		
Gondar town	219	43.45
Out of Gondar town	285	56.55
Had ANC in the index pregnancy		
Yes	483	96.22
No	19	3.78
TT vaccination in index pregnancy^a^		
Not vaccinated	20	4.08
TT one	36	7.35
TT two and above	434	88.57
Parity (number of births)		
I	259	51.39
II–IV	182	36.11
≥ V	63	12.50
Complication during index pregnancy (n = 157)		
Antepartum hemorrhage (APH)	33	6.55
Premature rapture of membrane (PROM)	66	13.10
Pregnancy-induced hypertension (PIH)	58	11.51
Previous bad obstetrics history^b^		
Yes	26	5.16
No	478	94.84
Type of pregnancy		
Singleton	404	80.16
Twin	100	19.84
Onset of labor		
Elective cesarean section	47	9.33
Spontaneous onset	414	82.14
Induced	43	8.53
Place of birth		
Home	19	3.77
Health center	116	23.02
Hospital	369	73.21
Mode of delivery		
Spontaneous vaginal delivery	328	65.08
Cesarean section	144	28.57
Instrument-assisted delivery	32	6.35

^a^Tetanus Toxoid, ^b^ Stillbirth or early neonatal death or intra-uterine fetal death

#### Characteristics of neonates who were admitted in NICU

Two hundred ninety-nine, (59.44%), neonates were males, and about 41% were preterm births. Before admission to NICU, quarters (25.6%) of neonates were resuscitated with bag and mask and nearly 46% were hypothermic at admission. Early onset neonatal sepsis was diagnosed among 59.33%, sepsis in general was diagnosed among 63.69% neonates and one neonate was diagnosed for both early and late onset sepsis during his/her stay in the unit (Table 2).

**Table 2:** Characteristics of neonates admitted in NICU of UoGCSH from January to December 2017, northwest Ethiopia (n = 504)

Characteristics	Frequency	Percent
Sex of the neonate		
Male	299	59.44
Female	204	40.56
Gestational age		
Preterm (< 37 weeks)	209	41.47
Term (≥ 37 weeks)	261	51.79
Post-term (≥ 42 weeks)	34	6.75
Weight for gestational age at admission		
Small	26	5.16
Appropriate	471	93.45
Large	7	1.39
Bag and mask resuscitation at birth		
Yes	129	25.60
No	375	74.40
Peri-natal asphyxia diagnosed at admission		
No perinatal asphyxia (PNA)	400	79.37
Yes	104	20.63
Hypothermia diagnosed at admission		
Yes	236	46.83
No	268	53.17
Hyper bilirubinemia diagnosed at admission		
Yes	30	5.95
No	474	94.05
Clinically diagnosed neonatal sepsis		
No	183	36.31
Early-onset sepsis	299	59.33
Late-onset sepsis	21	4.17
Combined (early and late)	1	0.20

#### Factors associated with neonatal sepsis

The multivariable logistic regression analysis revealed that intra-partum maternal fever found to be statistically significant predictor for both early-onset [AOR: 10.86, 95% CI (1.35–87.7)] and overall neonatal sepsis [AOR: 8.64, 95% CI (1.06–70.4)]. Neonates who were admitted during the dry and moist season from September to November were more likely to have both early-onset [AOR: 1.89, 95% CI (1.02–3.51)] and overall neonatal sepsis [AOR: 2.18, 95% CI (1.67–4.05)] as compared to June to August rainy season admissions. Moreover, neonates delivered vaginally were about 2 times [AOR: 2.22, 95% CI (1.24–4.32)] likely to have an early-onset and overall [AOR: 2.14, 95% CI (1.21–3.77)] sepsis. Likewise, neonates born before 37 complete weeks were likely to have early-onset [AOR: 5.03, 95% CI (3.04–8.33)] and overall [AOR: 4.03, 95% CI (2.43–6.71)] sepsis as compared to their counterparts (Table 3).

**Table 3:** Factors associated with neonatal sepsis among neonates admitted in NICU of UoGCSH from January to December 2017, northwest Ethiopia (n = 504)

Variables	Neonatal sepsis (overall sepsis)		AOR (95% CI)	Neonatal sepsis (EONS)		AOR (95% CI)
	Yes	No		Yes	No	
Had ANC						
Yes	306	179	1	287	198	1
No	15	4	1.44 (0.42, 4.93)	13	6	0.94 (0.30, 2.94)
History of APH						
Yes	27	6	1.79 (0.64, 5.01)	26	7	1.49 (0.56, 3.96)
No	294	177	1	274	197	1
Maternal fever during labor						
Yes	19	1	8.64 (1.06, 70.4)	19	1	10.86 (1.35, 87.7)
No	302	182	1	281	203	1
Duration of labor						
Zero h	25	22	1	25	22	1
1–12 h	188	116	1.08 (0.47, 2.49)	172	132	0.96 (0.41, 2.26)
> 12 h	108	45	2.19 (0.87, 5.51)	103	50	1.94 (0.76, 4.94)
Place of birth						
Home	16	3	3.90 (0.96, 15.79)	11	8	1.36 (0.43, 4.33)
Health center	87	29	1.78 (0.94, 3.38)	289	196	1.69 (0.89, 3.22)
Hospital	218	151	1			1
Season of birth						
Sep–Nov	85	34	2.18 (1.67, 4.05)	77	42	1.89 (1.02, 3.51)
Dec–Feb	74	47	0.92 (0.50, 1.68)	71	50	0.93 (0.51, 1.72)
Mar–May	93	57	1.31 (0.74, 2.32)	86	64	1.26 (0.71, 2.25)
Jun–Aug	69	45	1	66	48	1
Mode of delivery						
Spontanous vaginal delivery	231	93	2.14 (1.21, 3.77)	213	111	2.22 (1.24, 4.32)
Cesarean delivery	69	75	1	66	78	1
Instrument-assisted delivery	21	15	1.27 (0.53, 3.06)	21	15	1.52 (0.62, 3.71)
Gestational age at birth						
Preterm (< 37 weeks)	168	41	4.03 (2.43, 6.71)	165	44	5.03 (3.04, 8.32)
Term (37–41 weeks)	133	129	1	115	146	1
Post-term (≥ 42 weeks)	21	13	1.59 (0.69, 3.61)	20	14	1.65 (0.72, 3.80)
Did the baby cry at birth						
Yes	231	159	1	210	180	1
No	90	24	1.33 (0.49, 3.55)	90	24	1.84 (0.69, 4.88)
5th minute APGAR score						
Unknown	102	60	0.77 (0.43, 1.36)	83	79	0.50 (0.28, 0.90)
< 7	54	15	1.68 (0.75, 3.77)	54	15	1.47 (0.65, 3.30)
≥ 7	165	108	1	163	110	1
Bag and mask ventilated at birth						
Yes	103	26	1.91 (0.76, 4.78)	103	26	1.79 (0.73, 4.44)
No	218	157	1	197	178	1

### Discussion

This research determined the proportion of neonatal sepsis and identified factors associated with sepsis among neonates admitted in the NICU of the University of Gondar Comprehensive Specialized Hospital.

Accordingly, the proportion of EONS was 59.33%, LONS was 4.17%, and overall sepsis was 63.69%. Maternal intra-partum fever, the season of birth and admission, vaginal delivery and preterm gestational age at births increased the likelihood of overall sepsis and EONS.

The proportion of neonatal sepsis in general and early-onset sepsis, in particular, was 63.69% and 59.33% in this study in that order. This finding is slightly lower than previous studies done in Gondar 77.8% [17], and Shashemene 77.9% [12]. This small variation could be attributed to the large sample size in the current study. However, both findings could reflect that neonatal sepsis is the most prevalent morbidity among neonates in developing countries.

The presence of maternal fever in the intra-partum period increased the likelihood of neonatal sepsis by 9 and early-onset sepsis by 11 times as revealed in this study. This finding is in line with previously conducted studies. Likewise, the odds of having neonatal sepsis was 6 times higher among intra-partum fever cases in Mekelle city, Ethiopia [18] and 37 times in Karachi, Pakistan [19]. In addition, a study in Bangladesh demonstrated that intra-partum fever was a significant predictor of neonatal sepsis [20]. Studies reported that intra-partum fever is suggestive of maternal infections. So that, maternal infection frequently transmitted to the baby as ascending, via circulation or during passage through the birth canal and it usually causes early-onset sepsis [20, 21].

In this study, the season of birth and admission has shown a statistically significant association with the probability of being diagnosed for neonatal sepsis in this study. Neonates born and admitted to NICU in September to November had nearly 2 times increased odds of developing neonatal sepsis than June to August births. A study conducted in Canada also identified a significant seasonal variation in neonatal sepsis in which an increased infection during the summer months was observed [22]. Another study from Benin City, Nigeria showed a significantly higher proportion of sepsis among female neonates during the dry season [23]. The reason related to seasonal variation in neonatal sepsis is unclear. However, an assumption that is related to high temperature with moderate humidity of such season might create a favorable environment for microbial growth could be attributed to the association [24].

In our study, the mode of delivery illustrated a statistically significant association with neonatal sepsis. Neonates born vaginally had twice more odds of developing neonatal sepsis than cesarean births. Some studies showed a higher odds of neonatal sepsis among cesarean births [13, 25]. On the other hand, the vaginal canal is colonized by different microbial and believed to result in neonatal colonization. As revealed in a study done in Pakistan [26], significantly increased neonatal sepsis in repeatedly done vaginal examination case could a be a logical explanation for this finding.

Neonates who were born before 37 complete weeks had more than 4 times higher odds of infection compared with term births. Previous studies also identified a positive association of sepsis among preterm neonates. A similar association was noted by the studies conducted in Makassar [27], Jakarta, Indonesia [28], and Mexico [29] in which preterm neonates were more likely to have an infection. Manuck et al. study further identified that neonatal morbidity was most frequently occurred among preterm groups [30]. This susceptibility is mainly ascribed to the limited capacity of preterm neonates to defend against microorganisms [31].

In this study, neonatal sepsis remaining the leading cause of morbidity among younger infants. Maternal intra-partum fever, a season of birth and admission, vaginal delivery and preterm gestational age at births increased the likelihood of overall sepsis and EONS. Mainly intra-partum conditions contributed to neonatal sepsis. Thus, providing emphasis on associated factors in particular and universal safe obstetric care, in general, is recommended.

## Limitations of the study

Despite retrospective nature, this study was generated from a single hospital and its representativeness is limited. Thus, it is informative to conduct large scale prospective study which shall address the quality of obstetric and neonatal services.

## Data Availability

The datasets used and/or analyzed during the current study are available from the corresponding author on reasonable request.

## Abbreviations

ANC: antenatal care
EONS: early-onset neonatal sepsis
LONS: late-onset neonatal sepsis
NICU: neonatal intensive care unit
UoGCSH: University of Gondar comprehensive specialize Hospital
